# A commentary: Do specific gene risk variants for schizophrenia contribute similarly to the incidence of schizophrenia world-wide?

**DOI:** 10.1038/s41537-017-0031-7

**Published:** 2017-08-30

**Authors:** Lynn E. DeLisi

**Affiliations:** Department of Psychiatry, VA Boston Healthcare System, Harvard Medical School, Brockton, MA 02301 USA

In the 1980s there was extensive debate as to whether schizophrenia exists to the same extent in all cultures and geographic areas throughout the world (reviewed in ref. 1). While some support from that era exists for clusters of illness in different regions,^2, 3^ and a lack of schizophrenia in some remote parts of the world,^4^ other evidence was reported indicating that, overall, it occurs at relatively uniform rates throughout the world.^5, 6^ Since strongly Mendelian-inherited causes are not distributed uniformly, proponents for heredity to be the major underlying basis for schizophrenia wondered how the latter could be possible, unless the underlying genetic architecture of the disease is not Mendelian, but rather a consequence of numerous genes of small effect (e.g., see ref. 7). These polygenes, some of which could be more prevalent in different parts of the world, still when added together, could contribute to the great majority of illness and a uniform incidence of it world-wide. An alternative argument was proposed that a gene relevant to the origins of humanity itself, and thus present in all human beings, had the potential to cause psychosis in its extreme manifestation.^8^ However, neither of these theories could be clearly tested until recently, with the remarkable new advances in molecular genetics that led to large scale genome-wide association and sequencing studies. The field has not yet arrived at definitive answers, but is coming closer each year. It is now thought that there are both multiple common risk alleles of small affect, as well as rare variants of larger effect (such as copy number variations: CNVs) that contribute to the overall genetic architecture of schizophrenia on a population basis.

In 2007 the Psychiatric Genomics Consortium (PGC) was initiated, and rapidly became a growing network of international investigators (https://www.med.unc.edu/pgc). Samples were contributed to this consortium to conduct a large GWAS that currently includes approximately 100,000 individuals.^9^ Since most of the sample collections are biased toward heavy proportions of people with European ancestry, very little is known about whether the results can be generalized to Asian and other cultures world-wide. Despite this biologic uncertainty, the disorder called “schizophrenia” is remarkably similar clinically, spanning European and Asian countries, despite language and cultural differences, thus contributing to the notion that there must be a common underlying biological pathway to this disease. It is more similar than different, despite its individual heterogeneity within cultures. How this clinical similarity generalizes to the level of the gene is unknown.

In this set of three separate articles, we have solicited reviews of genetic data from prominent psychiatric genetic research groups in non-European, Asian populations (China, Japan and India), each with different genetic ancestry. Although large-scale GWASs have not been performed in these countries, as has within the PGC, nevertheless, some trends merge, which are not surprising. Some of the high-risk genes emerging from the PGC are also significant in these three populations, while others are novel and unique.

In the Chinese Han population, which consists of the majority of the Chinese people, susceptibility gene variants, such as within the major histocompatibility complex (*MHC*), microRNA 137, zinc finger protein 804 A, vaccinia related kinase 2, and arsenite methyltransferase, are shared with the European ancestry populations, and in fact some of these are also significant risk factors in the Japanese and India studies as well, implicating their likely relevance world-wide. Several CNVs identified in European populations also have been validated in the Han Chinese, including duplications at 16p11.2, 15q11.2-13.1, 7q11.23, and VIPR2, and deletions at 22q11.2, 1q21.1-q21.2, and *NRXN1*. On the other hand, the tetraspanin 18 or zinc finger protein 323 was found to harbor risk alleles that so far are unique to the Chinese Han population.

In Japan, studies have been published to confirm again some of the findings of the large European GWAS,^9^ such as in the MHC region, and also *CSMD1* and *GRM7*, but the studies are thus far too small to examine all the other possible candidates. Some of the reported risk CNVs have also been confirmed, but not yet others.

Similarly in India, aside from minimally replicating the chromosome 6p region finding within the MHC complex, other novel loci have been suggestive, although not reaching significance, such *NFKBIL1 and MICB* (immune response genes) and *AHI1* (Abelson helper integration site-1), which is a common helper provirus integration site for murine leukemias and lymphomas and also has been shown to be involved in neurodevelopment.

Thus, while these small initial studies from Asian populations at least partially confirm some, but not all of the European GWAS results, not surprisingly, there are other findings that are unique to these populations based on their population ancestry. More risk alleles are likely to be found to be both in common with other populations and also unique to each, specifically when much larger sets of samples are examined in each country. Regardless, it appears certain that all findings are likely to converge on a specific pathology in pathways underlying the biological basis for schizophrenia, and these pathways are likely to be uniform world-wide, such as in the immune system, and in brain development of key cortical structures (e.g., see ref. 10). However, if ultimately markers to predict relative risk or treatment efficacy and side-effects of medications are to be used clinically, the country of ancestral origin will need to be considered and the specific relevant markers examined.
